# Effects of catechin-enriched ion beverage intake on thermoregulatory function in a hot environment

**DOI:** 10.1007/s12576-018-0615-7

**Published:** 2018-04-23

**Authors:** Rumiko Nishimura, Naoki Nishimura, Satoshi Iwase, Masao Takeshita, Mitsuhiro Katashima, Yoshihisa Katsuragi, Motohiko Sato

**Affiliations:** 1 Department of Physiology, Aichi Medical University School of Medicine, 1-1 Yazako-Karimata, Nagakute, Aichi 480-1195 Japan; 2 Department of Sport Sciences, Nihon Fukushi University, 35-6 Aza-Egemae, Ooaza-Okuda, Mihama, Chita, Aichi 470-3295 Japan; 3 R&D, Development Research-Health Care Food Research, Kao Corporation, 2-1-3, Bunka, Sumida, Tokyo 131-8501 Japan

**Keywords:** Catechin-enriched ion beverage, Hypotonic beverage, Thermoregulatory function, Sweat rate, Skin blood flow

## Abstract

We examined the effect of intake of a catechin-enriched ion beverage (Cat-I) on the thermoregulatory response in a hot environment. Eight healthy men were exposed to a hot environment for 90 min at an ambient temperature of 35 °C (relative humidity: 75%) combined with lower leg water immersion at 40 °C. At that time, either Cat-I, an ion beverage (Ion), or mineral water (Placebo) was consumed at three points: (1) at the start of lower leg immersion, (2) at 30 min after immersion, and (3) at 60 min after immersion. In all conditions, tympanic temperature (Tty) increased gradually during lower leg water immersion. However, the rate of increase of Tty tended to be suppressed after 30 min. The effect of drinking Cat-I had a limited detection period of approximately 60–70 min, and the rate of sweating was clearly increased with Cat-I compared with Ion and Placebo. Cat-I also tended to decrease the body temperature threshold at which sweating was induced compared with Ion or Placebo. These findings suggest that Cat-I efficiently suppressed the increase of body temperature in a hot environment.

## Introduction

Green tea is a popular beverage in Japan, and it has been used as a medicine since ancient times in Asian countries. Tea catechins are a group of polyphenols contained in the leaves of green tea that exert antioxidant [1, 2], antiallergenic [3], antiatherogenic [4] and other pharmacological activities. They also boost energy expenditure [5]. Maki et al. [6] reported that the combination of intake of a high-concentration tea catechin beverage for 12 weeks and physical activity improved lipid metabolism. Tea catechins are also known to modulate dermal blood circulation and skin surface hydration [7, 8]. Heinrich et al. [8] demonstrated that intake of catechin beverages containing cocoa dietary flavanols for 12 weeks significantly enhanced skin blood flow compared with baseline. On the other hand, Dulloo et al. [9] reported that energy expenditure was significantly increased by a single intake of catechin-containing green tea. Heinrich et al. [8] suggested that catechin intake can promote beneficial thermoregulatory responses in humans by promoting heat dissipation in a hot environment. However, the effect of a single intake of a catechin-containing beverage is unclear.

Fluid intake is an important factor in preventing heat stroke, but the intake of only pure water may reduce the osmotic pressure of body fluids, leading to water diuresis, which sometimes causes spontaneous dehydration [10]. Therefore, the consumption of ion drinks that contain electrolytes and amino acids is recommended for hydration in a hot environment [11, 12]. These beverages are adjusted to be close to the osmotic pressure of body fluids in order to be absorbed more readily by the digestive system [13]. Many conventional ion drinks are intended to be drunk when playing sports or performing strenuous labor in a hot environment, so they include glucose as an energy source as well as water and electrolytes, which tend to be lost when sweating. However, there are reports that for hydration in a hot environment, hypotonic drinks suppress the decrease in plasma volume [14] and elevate serum osmolality more than isotonic drinks [14–16]. Hypotonic sports drinks, which have low levels of carbohydrates, are absorbed more rapidly than standard isotonic drinks and mineral water, and hypotonic sports drinks are considered to be an effective ergogenic aid for endurance performance [15]. However, the effectiveness of the intake of a combination of tea catechins and hypotonic beverages on human thermoregulatory functions, such as skin blood flow and sweating rate, which play important roles in heat dissipation, has not been reported.

In this study, we examined the effect of the intake of a tea catechin-enriched ion beverage (Cat-I) on thermoregulatory response during lower leg water immersion in a hot environment. We hypothesized that hypotonic Cat-I intake would aid human thermoregulatory function in a hot environment.

## Methods

Cat-I and mineral water (Placebo) were provided by Kao Co., Ltd. The ion beverage (Ion) used was manufactured by Otsuka Pharmaceutical Co., Ltd. from conventionally available drinking water (Table 1). The temperature of the drinks was set at 35 °C using a thermal insulation cabinet to match the environmental temperature in the study.

**Table 1:** Constituents of the catechin-rich ion beverage (Cat-I), ion beverage (Ion) and mineral water (Placebo)

	Cat-I	Ion	Placebo
mg/100 ml			
Sodium	34	49	1
Potassium	2.5	20	0.5
Carbohydrate	1.6	6.2	0
Osmotic pressure	162	324	2
Tea catechin	121	0	0

### Study design

Participants were 8 healthy men [age: 26 ± 8 years; body mass index: 22.0 ± 2.9% (mean ± standard deviation)]. After receiving a sufficient explanation of the study, all participants provided written informed consent. The protocol of the study was approved by the institutional review board of Aichi Medical University. The study was conducted in accordance with the principles of the Declaration of Helsinki.

**Fig. 1:**
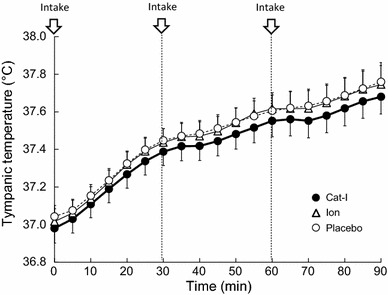
The time course of tympanic temperature during lower leg water immersion with intake of Cat-I, Ion and Placebo. *Arrows* indicate the timing of beverage intake. Values are mean ± SD

No participants had a habit of consuming caffeine- or catechin-containing beverages, but they were requested to abstain from caffeinated beverages and alcohol for at least 12 h before the experimental sessions. They were required to fast after 20:30 the night before the experiment. On the day of the experiment, they were given 400 kcal jelly (Calorie Mate Jelly; Otsuka Pharmaceutical Co., Ltd., Otsuka, Japan) and then requested to drink 500 ml mineral water at 07:00. Then, they drank 500 ml mineral water by noon, and had a light meal at 12:30 with 250 ml barley tea and 350 ml mineral water. They were requested to finish the mineral water by 14:30 and to fast until the experiment started. After they arrived at the laboratory at 16:30, they entered an artificial climate-controlled chamber (pre-room) with an ambient temperature of 25 °C and 50% relative humidity. They changed into a T-shirt and swimsuit, and 20 ml blood was drawn and their weight was measured. Then, a tympanic temperature (Tty) sensor, skin temperature sensors (arms, upper arms, thighs, and lower legs), a blood pressure cuff, a laser Doppler flowmetry probe for monitoring skin blood flow (upper arm), and a capsule for evaluating local sweating (upper arm) were used to take measurements of the participants. They sat on a chair for 10 min, and then moved to the climate chamber set to a mid-summer environment (ambient temperature: 35 °C; relative humidity: 75%), and immersed their lower legs in a sitting position into water at a temperature of 40 °C for 90 min. The participants started drinking immediately when they immersed their legs; we termed this point “time 0” for the analysis (Fig. 1). Cat-I, Ion, or Placebo was consumed 3 times: (1) when lower leg water immersion was started, (2) at 30 min after immersion, and (3) at 60 min after immersion (Table 1). Fluid intake at each time point was body weight (kg) × 4 (ml), and the participants were requested to consume the drink within 3 min because it is well known that diuretic action is induced when a large amount of liquid is ingested all at once. After lower leg water immersion for 90 min, the participants were moved back to the pre-room. Then, 20 ml blood was drawn again.

### Measurements

Tty, local sweating rate, and skin blood flow were measured continuously. Tty was measured using a thermistor (ST-21S; Sensor Technica, Aichi, Japan) inserted into the external meatus. The ear orifice was filled with a mass of cotton to fix the thermistor probe in place and to avoid the effect of ambient temperature. The local sweating rate on the upper arm was recorded using the ventilated capsule method with a capacitance hygrometer (HMI-23; Vaisala, Helsinki, Finland). The sweat capsule, covering an area of 8 cm^2^, was ventilated with dry nitrogen gas at a flow rate of 1.5 l/min, and humidity change of the effluent air was sensed by calibration with 1 μl pure water injection. Skin blood flow was measured by means of laser Doppler flowmetry (ALF-21; Advance, Tokyo, Japan) with a probe attached to the upper arm. Blood pressure was measured every 30 min at the left-upper arm using an automated sphygmomanometer by the oscillometric method. Blood samples were drawn in the pre-room before and after lower leg water immersion, and hemoglobin concentration, hematocrit, and plasma sodium level were determined. ΔPV was calculated using the Dill & Costill formula [17].

### Data analysis

All data are expressed as the mean ± standard deviation. Statistical significance between beverage conditions and Placebo was calculated using two-way analysis of variance followed by Greenhouse–Geisser or Huynh–Feldt multiple comparison tests. *P* values < 0.05 were considered significant.

## Results

**Fig. 2:**
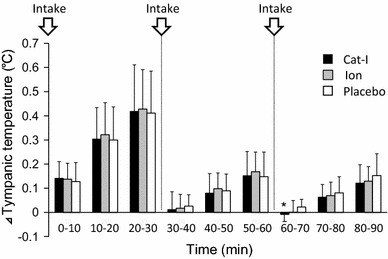
Change of tympanic temperature during lower leg water immersion. Data are shown as average values in every 10 min. The change of temperature was calculated as increase of temperature compared with time of intake of beverages. Thus, in 0- to 30-min period, temperature was compared with point of “0” in figure; in 30–60 min, temperature was compared with point of “30” in Fig. 1; in 60–90 min, temperature was compared with point of “60” in Fig. 1. *Arrows* indicate the timing of beverage intake. Values are mean ± SD. *p < 0.05 compared with mineral water

We first examined the effects of Cat-I, Ion, and Placebo on increases in Tty, which is a valid indicator of core temperature, during lower leg water immersion (Fig. 1). Although Tty increased gradually during lower leg water immersion, its rate of increase after drinking water tended to be suppressed for all beverages, although the temperature of the beverage consumed during lower leg water immersion was 35 °C, which was the same temperature as the environment. Then, to examine the suppression of Tty increase by Cat-I intake compared with Ion and Placebo, the average rate of change in Tty every 10 min after drinking a test beverage during lower leg water immersion was compared between each beverage (Fig. 2). For the mean values at 60–70 min, Cat-I intake led to a significant decrease in the increase of Tty (*P* < 0.05) compared with Placebo intake. Although the suppression of the increase of Tty by drinking before lower leg water immersion was unclear, it became obvious after repeated drinking.

**Fig. 3:**
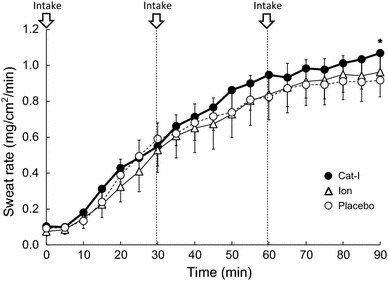
The time course of upper arm sweat rate changes during lower leg water immersion with intake of Cat-I, Ion and Placebo. Arrows indicate the timing of beverage intake. Values are mean ± SD. *p < 0.05 compared with mineral water

**Fig. 4:**
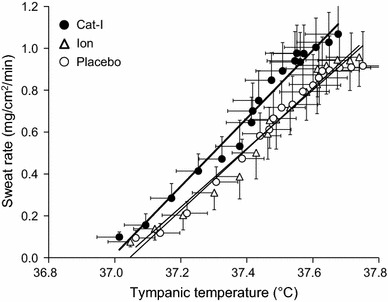
Relationship between sweat rate and tympanic temperature during leg water immersion with intake of Cat-I, Ion and Placebo

It is well known that an increase in sweating rate and skin blood flow leads to the suppression of Tty increase by heat dissipation. We examined the effect of Cat-I, Ion, and Placebo on the increase in upper arm sweating rate during lower leg water immersion, which is the most effective indicator of heat dissipation (Fig. 3). Upper arm sweating increased gradually during lower leg water immersion, but increased more with the intake of Cat-I compared with Ion or Placebo. After 90 min of lower leg water immersion, the sweating rates were 1.06 ± 0.38 mg/cm^2^/min with Cat-I, 0.98 ± 0.39 mg/cm^2^/min with Ion, and 0.92 ± 0.46 mg/cm^2^/min with Placebo. Cat-I induced a significantly higher sweating rate (*P* < 0.05) than Placebo. The threshold for the onset of sweating was lower for Cat-I intake than for Ion and Placebo, and the sweating rate for the same Tty was higher for Cat-I than with Ion and Placebo, especially at higher Tty (Fig. 4). Cat-I also tended to decrease the body temperature threshold for the onset of sweating compared with Ion and Placebo.

**Fig. 5:**
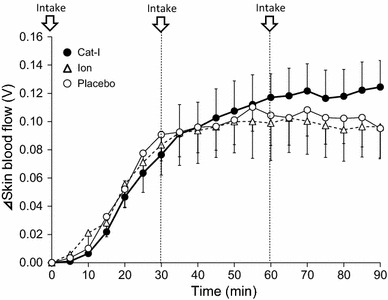
The time course of upper arm skin blood flow changes during lower leg water immersion with intake of Cat-I, Ion and Placebo. *Arrows* indicate the timing of beverage intake. Values are mean ± SD

Figure 5 shows the effect of Cat-I, Ion, and Placebo on increased skin blood flow during lower leg water immersion, which is an indicator of heat dissipation. Upper arm skin blood flow increased rapidly for up to 30 min of lower leg water immersion for all beverages. After that, the increase in skin blood flow for Cat-I seemed to be slower than for the other beverages but continued for longer.

**Fig. 6:**
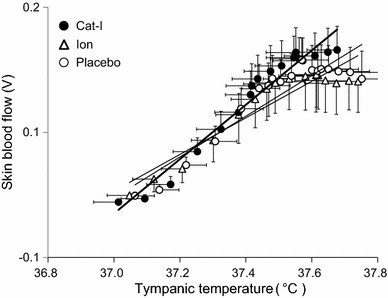
Relationship between skin blood flow and tympanic temperature during leg water immersion with intake of Cat-I, Ion and Placebo

Figure 6 shows the relationship between skin blood flow and Tty during lower leg water immersion. The slope of the regression line was steeper for Cat-I than for the other beverages. Especially, when Tty was high, skin blood flow was higher for Cat-I than for Ion and Placebo.

Table 2 shows the plasma properties and blood pressure before and after lower leg water immersion. The plasma properties showed no significant differences among the different beverages. There was no significant difference in the increase of systolic blood pressure after lower leg water immersion between Cat-I (104.9 ± 8.4%), Ion (106.5 ± 6.8%), and Placebo (104.3 ± 5.3%). Diastolic blood pressure decreased after lower leg water immersion, but it was not significantly different between Cat-I (83.9 ± 13.5%), Ion (93.1 ± 13.7%), and Placebo (84.6 ± 14.1%).

**Table 2:** Plasma properties and blood pressure before and after leg water immersion with intake of Cat-I, Ion and placebo

	Cat-I			Ion			Placebo		
	Before	After	%Δ	Before	After	%Δ	Before	After	%Δ
[Na^+^] (mosmol/kgH_2_O)	140.4 ± 1.2	139.3 ± 1.7	99.2 ± 0.9	140.8 ± 1.0	140.3 ± 1.7	99.6 ± 1.3	140.5 ± 0.9	139.4 ± 1.1	99.2 ± 0.8
Osmolality (mosmol)	281.3 ± 2.1	279.8 ± 3.8	99.5 ± 1.1	280.8 ± 3.0	280.9 ± 2.6	100.1 ± 1.2	280.9 ± 1.7	278.6 ± 2.2	99.2 ± 0.8
Hb	15.5 ± 1.0	15.8 ± 1.1	104.4 ± 2.8	15.5 ± 1.3	15.9 ± 1.3	104.4 ± 3.7	15.4 ± 0.9	15.9 ± 1.0	105.1 ± 2.6
HcT	47.6 ± 1.9	48.3 ± 3.0	103.7 ± 2.7	47.2 ± 2.8	48.3 ± 3.5	104.3 ± 4.3	47.2 ± 2.2	48.0 ± 3.0	104.0 ± 3.8
ΔPV			− 7.4 ± 4.9			− 7.5 ± 6.6			− 8.1 ± 5.2
SBP (mmHg)	106.0 ± 8.5	112.5 ± 13.5	104.9 ± 8.4	106.5 ± 13.8	113.8 ± 12.1	106.5 ± 6.8	105.8 ± 7.2	111.75 ± 10.2	104.3 ± 5.3
DBP (mmHg)	68.0 ± 8.8	60.0 ± 16.5	83.9 ± 13.5	68.0 ± 8.8	60.0 ± 16.5	93.1 ± 13.7	66.0 ± 11.5	58.35 ± 17.3	84.6 ± 14.1

Values are the mean ± SD of 8 subjects. ΔPV was calculated using the formula of Dill & Costill. SBP and DBP values indicate the start (before) and end (after) of lower leg immersion

## Discussion

The present study demonstrated the potential effects of Cat-I on thermoregulatory function. First, Cat-I intake significantly increased (*P* < 0.05) the sweating rate of the upper arm compared with Placebo intake during lower leg water immersion in a hot environment (Fig. 3). Second, the increase in skin blood flow due to Cat-I intake at a high body temperature during lower leg water immersion was higher than after Ion and Placebo intake (Fig. 5). As a result, the degree of Tty increase was suppressed due to Cat-I intake to a limited extent (Figs. 1, 2). The temperature of the beverages was controlled to be the same as the ambient temperature to minimize any potential cooling effect. This novel study revealed that Cat-I intake in a hot environment was useful for lowering core temperature and may help to prevent heat stroke in hot environments.

Even in a hot environment, if environmental temperature is lower than body temperature, an increase in the sweating rate and skin blood flow leads to a direct increase in heat dissipation. In the present study, the sweating rate was significantly higher with Cat-I intake than with Placebo (Fig. 3), and the sweating rate at the same Tty was higher with Cat-I intake than with the other beverages (Fig. 4). In addition, the increase in skin blood flow was higher with Cat-I than with Ion and Placebo (Fig. 5). These maximum effects of catechins on the thermoregulatory system appeared at approximately 60 min after ingestion in the present study. According to previous studies, it takes 60–90 min after catechin ingestion for the concentration of catechins to reach its maximum [18, 19], which appears to be consistent with the time observations in our study. The effect of catechins on the change of temperature appeared at a late time point in the study period, which may reflect a time lag for catechins to reach an effective concentration.

Many reports have examined the physiological and pharmacological effects of tea catechins contained in the hypotonic beverage used in the present study [1, 2, 5, 6, 20]. Although the effect of catechin-containing beverage intake on sweating has not been reported, an association between cutaneous vasodilation and the activation of sympathetic sudomotor nerves has been observed [21, 22]. Sympathetic neural control of skin blood flow during heat stress includes the sympathetic active cutaneous vasodilator system and nitric oxide. Epicatechin, a major component of tea catechins, is associated with nitric oxide signaling, and it has been shown to contribute to the skin vasodilator response during whole body heating [23, 24]. Dietary constituents including catechins were also reported to improve cutaneous and subcutaneous blood circulation and skin hydration [7, 8]. Heinrich et al. [8] demonstrated that the intake of catechin beverages containing cocoa dietary flavanols for 12 weeks significantly enhanced skin hydration and skin blood flow compared with baseline.

Recently, sensory nerves have been revealed to play an important role in cutaneous vasodilation during local skin heating [25]. Additionally, temperature-sensitive transient receptor potential vanilloid type 1 (TRPV1) channels located in sensory nerve terminals have also been reported to play an important role in the cutaneous vasodilator response to local heating [26]. Tea catechins are known to activate transient receptor potential ankyrin type 1 (TRPA1) and TRPV1 in sensory neurons in intestinal enteroendocrine cells [27, 28]. Thus, the greater increase in skin blood flow with Cat-I intake than with Ion or Placebo might be related to the activation of TRP channels. Furthermore, the administration of TRPA1 agonists caused increases in heart rate and blood pressure that may have resulted from elevated sympathetic nervous activity [29]. For these reasons, we considered that Cat-I intake increased skin blood flow and sweat rate.

For fluid intake, especially in hot environments, ion drinks are considered to be optimal because they: (1) have a high transit rate from the stomach to the small intestine; (2) have a high absorption rate in the intestinal tract; and (3) have a rapid recovery capacity for blood volume. Therefore, the intake of ion drinks that include electrolytes, carbohydrates, and amino acids is recommended for preventing heat stroke [11, 12, 15]. In addition, it has been reported that the intake of ion beverages is effective for recovery from dehydration and high blood viscosity due to sweating during bathing [16].

Many conventional ion beverages are drunk when playing sports or performing strenuous labor in a hot environment, so they include carbohydrates as an energy source as well as water and electrolytes, which are lost when sweating. Costill et al. [30] suggested that the ingestion of a beverage containing electrolytes and carbohydrates facilitates the recovery of plasma volume loss after dehydration more than the consumption of mineral water. Kamijo et al. [11] reported that carbohydrates in a diluted electrolyte solution enhanced renal Na^+^ reabsorption during thermal- and exercise-induced dehydration in young male participants, and that insulin is possibly involved in this enhancement. However, these ion beverages have a very sweet taste, which might result in a smaller volume being drunk during exercise in hot environments. The guidelines of the American College of Sports Medicine [31] state that factors for improving the taste of beverages include sodium content and a water temperature of 15–20 °C. Furthermore, guidelines for the prevention of heat stroke by the Japan Amateur Sports Association [32] recommended drinking water at a temperature of 5–15 °C. However, it is not always possible to maintain drinking water at a cold temperature in the field. Therefore, it should be noted that Cat-I intake at 35 °C facilitated sweating, increased skin blood flow, and suppressed the increase of body temperature more than the other beverages examined in the present study.

It was unexpected that there was no difference in thermoregulatory mechanisms such as sweating rate and skin blood flow between Placebo and Ion intake in this study. However, hydration was more important than the carbohydrates contained in Ion because the study conditions did not include exercise. In addition, the participants actively ingested the beverages every 30 min.

In summary, Cat-I intake facilitated sweating of the upper arm during lower leg water immersion in a hot environment, exhibiting a significantly higher rate of sweating (*P* < 0.05) than the Placebo beverage, which helped to regulate body temperature. In addition, the increase in skin blood flow due to Cat-I intake at a high body temperature during lower leg water immersion was higher than with Ion or Placebo intake. Therefore, increases in body temperature were suppressed by Cat-I intake, which facilitates heat dissipation. These findings on sweating facilitation and vasodilatory heat dissipation indicated that Cat-I intake in a hot environment would be useful in helping to prevent heat stroke.

## Limitations

The present study revealed that Cat-I was effective for increasing skin blood flow and sweating rate, as indicators of thermoregulatory function in a hot environment. However, it is difficult to exclude the effect of isotonic catechin beverage or hypotonic beverage intake on thermoregulatory responses in this pilot study. Chronic ingestion of catechin or hypotonic beverage has several beneficial effects in terms of plasma volume, osmolality, and lipid metabolism, whereas a single oral ingestion of catechin beverage in this study did not alter the blood properties associated with heat stress. Therefore, repeated intake of a catechin beverage is required to realize all the potential beneficial effects.

## Abbreviations

BMI: Body mass index (%)
Cat-I: Catechin-enriched ion beverage
ΔPV: Delta plasma volume
Hb: Hemoglobin concentration
Hct: Hematocrit (%)
Ion: Ion beverage
NO: Nitric oxide
*T*_max_: Maximum concentration time
TRPA1: Transient receptor potential ankyrin type1
TRPV1: Transient receptor potential vanilloid type1
Tty: Tympanic temperature (°C)
